# Advantages and Challenges in Using Telehealth for Home-Based Palliative Care: Protocol for a Systematic Mixed Studies Review

**DOI:** 10.2196/22626

**Published:** 2021-05-21

**Authors:** Simen A Steindal, Andréa Aparecida Goncalves Nes, Tove E Godskesen, Susanne Lind, Alfhild Dhle, Anette Winger, Jane Österlind, Fredrik Solvang Pettersen, Heidi Holmen, Anna Klarare

**Affiliations:** 1 Lovisenberg Diaconal University College Oslo Norway; 2 Department of Health Care Sciences Ersta Sköndal Bräcke University College Stockholm Sweden; 3 Centre for Research Ethics & Bioethics Uppsala University Uppsala Sweden; 4 Department of Health Care Sciences Palliative Research Centre Ersta Sköndal Bräcke University College Stockholm Sweden; 5 Department of Nursing and Health Promotion Faculty of Health Sciences Oslo Metropolitan University Oslo Norway; 6 Clinical Psychology in Healthcare, Department for Women’s and Children’s Health Uppsala University Uppsala Sweden

**Keywords:** eHealth, health care technology, home-based, palliative care, review, systematic mixed studies review, telemedicine

## Abstract

**Background:**

Given the increasing number of people in need of palliative care services and the current health care professional workforce strain, providing equitable, quality palliative care has become a challenge. Telehealth could be an innovative approach to palliative care delivery, enabling patients to spend more time or even remain at home, if they wish, throughout the illness trajectory. However, no previous systematic mixed studies reviews have synthesized evidence on patients’ experiences of the advantages and challenges of telehealth for home-based palliative care.

**Objective:**

The aim of this systematic mixed studies review is to critically appraise and synthesize findings from studies that investigated patients’ use of telehealth in home-based palliative care with a focus on the advantages and challenges experienced by the patients.

**Methods:**

This article describes the protocol for a systematic mixed studies review with a convergent design. The reporting will be guided by the PRISMA (Preferred Reporting Items for Systematic Reviews and Meta-Analyses) statement. A systematic search was performed in eight databases for studies published from January 2010 to June 2020. The search will be updated in 2021. Pairs of authors will independently assess eligibility, extract data, and assess methodological quality. The data will then be analyzed using thematic synthesis.

**Results:**

We describe the rationale and design of a systematic mixed studies review. The database searches were performed on June 25, 2020. Assessment of eligibility and further steps have not yet been performed. Results are anticipated by August 2021.

**Conclusions:**

Following the ethos of patient-centered palliative care, this systematic mixed studies review could lead to recommendations for practice and policy, enabling the development and implementation of telehealth applications and services that align with patients’ preferences and needs at home.

**International Registered Report Identifier (IRRID):**

PRR1-10.2196/22626

## Introduction

### Background

Palliative care is an approach that aims to improve the quality of life of patients and their families facing problems associated with life-threatening illness, regardless of diagnosis [1]. Historically, palliative care has been strongly associated with end-of-life care and cancer. Today, however, providers strive to introduce palliative care earlier in the illness trajectory [2] and to broaden the scope to include, for example, neurological diseases, lung conditions, frailty, cognitive impairment, and the presence of multiple comorbidities [3]. Recently, the need to change from disease-centered to patient-centered care has been highlighted [2,4,5]. This shift compels health care professionals to become more responsive to individual patient preferences, needs, and values, in addition to ensuring that patients’ values guide their clinical decision-making. Patients receiving palliative care often want to spend as much time as possible in their homes, and some even want to die at home [6,7].

Early integration of home-based palliative care for patients with life-threatening illness may improve patient and family satisfaction; improve patient quality of life; improve symptoms such as pain, fatigue, and nausea; and reduce aggressive treatment at the end of life; in addition to reducing hospital length of stay and hospitalization [8]. Feeling secure seems to be a core mechanism of palliative care enabling patients to stay at home [9,10]. While being cared for by a present and available team, patients may feel more supported and that someone shares the responsibilities of managing their illness [10].

Current ongoing circumstances present challenges in providing palliative care. Recently, the COVID-19 pandemic has resulted in an emphasis on telehealth. As telehealth applies to palliative care, it could potentially overcome obstacles when physical distancing requirements and lockdowns would otherwise limit access, subsequently increasing isolation and suffering [11,12]. In addition, it appears unclear how health care professionals are to treat the increased number of patients with palliative care needs [13]. Furthermore, there are concerns regarding future workforce strain in palliative care, both due to population growth and aging, and because a large proportion of the health care professionals retire or leave this area of practice, resulting in insufficient numbers to cover the shortfall [14]. The United Nations Agenda 2030 Sustainable Development Goal 3 highlights the right to health and well-being, with an emphasis on access to quality health care services [15]. Taken together, these challenges call for innovation and change in models of home-based palliative care delivery. Telehealth could be an important service addition to deliver high-quality home-based palliative care, enabling patients to spend as much as time as possible at home. Telehealth may empower patients to manage their illness, improve patient quality of life, decrease hospital admissions, and improve access to home care palliative care services [16,17].

Telehealth is defined as “the provision of healthcare remotely by means of a variety of telecommunication tools” [18]. Patients receiving palliative care and their next of kin have expressed that telehealth should be offered as a supplement to exiting services and should be used based on personal choices [19]. Health care professionals may be reluctant to use telehealth in palliative care. Previous research has identified concerns about increased focus on the patients’ physical problems, and that telehealth could have a negative effect on contact with patients [20,21].

The technology acceptance model (TAM) [22] is a theoretical model that has been used in studies regarding acceptance of technology in health care [23-25]. The goal of using the TAM theory is to elaborate the use of technology and users’ behaviors, in addition to better understanding acceptance of technology. Usability and ease of use are commonly associated with the user’s acceptance of the technology.

Systematic reviews have examined the use of telehealth in pediatric palliative care [26,27]. One systematic review of qualitative and quantitative studies examined existing information and communication technology (ICT) systems intended to support pain management in patients with cancer who were receiving palliative care. This review categorized these ICT systems as emergent; however, none of them had been implemented yet in clinical practice. The studies included were not limited to patients’ experiences of using these ICT systems in the home setting [28]. Two other systematic reviews have identified mobile apps developed and used in palliative care; however, neither of these reviews investigated patients’ experiences using these apps [29,30]. Systematic reviews have also examined the effectiveness of telehealth interventions and information needs in palliative care [31], as well as patient-reported outcomes such as quality of life, symptom management, and satisfaction [32]. Capurro et al [31] found that management of pain and other burdensome symptoms, and care in general, were the most frequent information needs in palliative care.

Another systematic review examined the use of telehealth in the monitoring of patients with chronic diseases at home to consider what could be adapted for patients receiving palliative care. However, the included studies were not limited to patients in a palliative care trajectory, nor did the results address the patients’ experiences of using telehealth at home [33]. Furthermore, an integrated review examined the use of video consultations in palliative care based on the views of patients, relatives, and health care professionals. The results suggested that video consultation is feasible in palliative care when used for communication between patients, relatives, or health care professionals, and for clinical assessments and symptom management. An important limitation with this review was that only one person performed the screening and data extraction [34].

### Why This Review is Needed

Recently, our group published a scoping review of patients’ experiences of using telehealth in palliative home care [35]. The results indicated that telehealth was easy and effortless to use, improved access to health care professionals at home, and enhanced patients’ feelings of safety and security. However, due to the scoping review design, the methodological quality of the included studies was not appraised, and the results of the included studies were grouped and not synthesized. Consequently, robust inferences and recommendations for policy and practice cannot be drawn or stated from the results. Another limitation was that 11 of the included studies were published before 2010, while studies on telehealth in palliative care have increasingly been published in the last 2 years. Furthermore, previous reviews have highlighted negative aspects of telehealth in general [17,36]. However, our review found that future systematic reviews should highlight the thus far neglected negative aspects of telehealth in palliative care [35].

Performing a scoping review is regarded as helpful in determining the value of undertaking a full systematic review [37]. Accordingly, based on this initial review, we conclude that the rationale and feasibility for a full systematic review using a systematic mixed studies review design is evident. To our knowledge, no systematic mixed studies reviews of primary research have synthesized evidence on patients’ experiences of the advantages and challenges using telehealth in home-based palliative care. Such a review could enable a comprehensive and rich understanding of the complex interventions and phenomena [38] occasioned by innovations such as telehealth for palliative care. Consequently, this systematic mixed studies review aims to critically appraise and synthesize findings from studies that investigated patients’ use of telehealth in home-based palliative care by answering the following research question: What do patients experience as the advantages and challenges of using telehealth in home-based palliative care?

## Methods

### Design

This systematic mixed studies review will employ a convergent design [38]. Studies will be included irrespective of their study design, and results from the included studies will be integrated using qualitative data transformation techniques. The PRISMA (Preferred Reporting Items for Systematic Reviews and Meta-Analyses) statement will guide the reporting of the review [39].

### Eligibility Criteria

Inclusion and exclusion criteria are described in Table 1. Included studies are limited to those published in peer-reviewed journals. Consequently, publications such as PhD theses will be excluded.

**Table 1:** Inclusion and exclusion criteria.

Category	Inclusion criteria	Exclusion criteria
Type of studies	Any type of quantitative, qualitative, or mixed methods study on the phenomenon of interest published in peer-reviewed journals	Any type of review, PhD thesis, conference abstracts, editorials, comments, or letters
Period	From January 1, 2010 to the updated search	Before January 1, 2010 and after the updated search
Language	Portuguese, Spanish, Danish, Norwegian, Swedish, or English	All other languages
Participants	Home-based patients aged 18 years or older in a palliative care trajectory, regardless of diagnosis	Patients 17 years or younger; not in a palliative care trajectory; or using telehealth in a nursing home, hospice, or hospital setting
Phenomenon of interest	Home-based patients’ experience of using telehealth with follow-up from health care professionals	Home-based patients’ experience of using telehealth without follow-up from health care professionals; or patients’ experience of using telehealth in a hospital, nursing home, or hospice
Outcomes	Patients’ subjective and objective outcomes	Proxy-reported outcomes

### Search Strategy

A systematic search was performed using the databases CINAHL, EMBASE, Medline, PsycINFO, Web of Science, Literature in the Health Sciences in Latin America and the Caribbean (LILACS), the Cochrane Central Register of Controlled Trials (CENTRAL), and Allied and Complementary Medicine (AMED) on June 25, 2020. The search strategy was built in Medline by FP, an experienced research librarian, and SS using text words and subject headings adopted for each of the databases used (see Multimedia Appendix 1). A second research librarian critically reviewed the search strategy. The search will be updated in 2021, approximately 2 months prior to submission of the manuscript for publication. We will contact authors of relevant conference abstracts to clarify whether the results have been published in a peer-reviewed journal. A manual search will be performed to screen the reference lists of the included papers and JMIR journals, as well as identifying references that cited the included articles after publication

### Study Selection

The identified publications were imported to EndNote for removal of duplicates. Rayyan QCRI will be used to facilitate storage, organization, and blinding of the identified publications [40]. Pairs of reviewers will independently assess whether titles, abstracts, and full-text publications meet the eligibility criteria. If there is any doubt about whether a publication meets these criteria, an additional reviewer will perform an independent assessment and discussions to reach negotiated consensus will take place [41].

### Appraisal of Methodological Quality

The methodological quality of the included studies will be independently appraised by pairs of reviewers using the relevant Johanne Briggs Institute critical appraisal tools based on the study design. If there is any conflict among the reviewers, a third reviewer will perform an independent appraisal and discussions to reach negotiated consensus will take place [41].

### Data Extraction

Data will be extracted from the included papers using a standardized data collection form by pairs of reviewers independently. The following data will be included: authors, year of publication, country of origin, aim of the study, study population and sample size, theoretical framework for the telehealth intervention, telehealth application, design and methods, and findings related to the research questions of the review.

### Data Synthesis

Data from the results section of the included papers will be extracted independently by pairs of reviewers. Results from studies that will include qualitative, quantitative, and mixed methods data will be transformed into a qualitative format [38]. Numerical data presented in tables and figures will be described with words. This will be supported by the authors’ description of the results from the results section of the included papers.

The data material will be analyzed using inductive thematic synthesis [38,42], which has previously been used in systematic mixed studies reviews with a convergent design [26,43]. NVivo (version 12) will be used to facilitate the storage and synthesis of data.

The extracted data material will be read several times to obtain an understanding of the material as a whole. The data material will be coded line by line according to its content and meaning. Text that has a code applied will be examined to check the consistency of interpretation and whether additional coding will be needed. Based on similarities and differences between the codes, the codes will be sorted into descriptive themes closely matching the results of the included studies. To generate analytical themes, the descriptive themes will be interpreted and abstracted, guided by the research question [42,44]. This will enable the analysis to go beyond the content of the included papers. The first author will be responsible for analyzing the data and developing codes and themes. The second author and the last author will read the data material and participate in discussions regarding the emerging codes and themes. The final themes will be decided by consensus among all authors. This could facilitate competing interpretations and thereby enhance credibility, dependability, and reflexivity.

## Results

We introduced the rationale and design of a systematic mixed studies review to critically appraise and synthesize findings to answer our research question: What do patients experience as the advantages and challenges when using telehealth in home-based palliative care? The database searches were performed on June 25, 2020, which identified 15,993 publications. After removal of 6799 duplicates, we will screen titles, abstracts, and full text of 9194 publications, in addition to manual searches and contacting researchers in this field. Results are anticipated by August 2021.

## Discussion

The results will be discussed in light of the TAM owing to its importance of understanding what influences patients’ acceptance of technology. Our review could contribute recommendations for practice and policy, enabling the implementation of patient-centered telehealth services that align with patient preferences, needs, and values.

## Appendix

Multimedia Appendix 1 Search strategy used in Medline.

## Abbreviations

ICT: information and communications technology
TAM: technology acceptance model
